# Live cell division dynamics monitoring in 3D large spheroid tumor models using light sheet microscopy

**DOI:** 10.1186/1747-1028-6-22

**Published:** 2011-12-12

**Authors:** Corinne Lorenzo, Céline Frongia, Raphaël Jorand, Jérôme Fehrenbach, Pierre Weiss, Amina Maandhui, Guillaume Gay, Bernard Ducommun, Valérie Lobjois

**Affiliations:** 1 Université de Toulouse; ITAV-UMS3039, F-31106 Toulouse, France; 2 CNRS; ITAV-UMS3039, F-31106 Toulouse, France; 3 Université de Toulouse; IMT-UMR5219, F-31062 Toulouse, France; 4 CNRS; IMT-UMR5219, F-31062 Toulouse, France; 5 CHU de Toulouse; F-31059 Toulouse, France

## Abstract

**Background:**

Multicellular tumor spheroids are models of increasing interest for cancer and cell biology studies. They allow considering cellular interactions in exploring cell cycle and cell division mechanisms. However, 3D imaging of cell division in living spheroids is technically challenging and has never been reported.

**Results:**

Here, we report a major breakthrough based on the engineering of multicellular tumor spheroids expressing an histone H2B fluorescent nuclear reporter protein, and specifically designed sample holders to monitor live cell division dynamics in 3D large spheroids using an home-made selective-plane illumination microscope.

**Conclusions:**

As illustrated using the antimitotic drug, paclitaxel, this technological advance paves the way for studies of the dynamics of cell divion processes in 3D and more generally for the investigation of tumor cell population biology in integrated system as the spheroid model.

## Background

Cell proliferation deregulation is a hallmark of tumor cells. The knowledge of the mechanisms of regulation of cell cycle control and cell proliferation is fundamental to a better understanding of the consequences of their misregulation in tumorigenesis, as well as to manipulate them in cancer therapy. The study of these mechanisms already contributed to a large extent to the improvement of targeted therapies. Nevertheless, most of these works suffer a major limitation that might account for the often-observed failure of new therapeutic strategies. Indeed, most, if not all of the studies performed so far relied on in vitro analyses of rapidly growing cancer cell lines in monolayer. These 2D models do not take into account tissue heterogeneity, cellular interactions and tumour microenvironment that have been shown to be of major relevance in tumour development [1,2]. Cell cycle control mechanisms are also dependent on cell-cell and cell-extracellular matrix interactions. For example, the orientation of the future cleavage plane at the mitosis is dependent on interaction of the cytoskeleton with intrinsic cortical factors but also with extrinsic cues including cell shape, cell-cell interactions and cell adhesion with extracellular matrix[3,4]. Therefore, studying mitosis ongoing in a 3D integrated cellular context would be of great interest.

Multicellular tumor spheroids (MCTS) generated from cancer cells are attractive models to study cancer proliferation in 3D. Indeed these 3D complex multicellular systems reproduce cell-cell and cell-matrix interactions as found in solid tumors [5]. Moreover, MCTS can grow to diameters of several hundred micrometers, progressively developing a gradient of proliferating cells similar to that found in non-vascularised micro-regions of a tumor: dividing cells are located in the outer layers and quiescent cells are located more centrally in regions that are hypoxic and receive few nutrients[6,7].

Despite our extensive knowledge of the molecular mechanisms that control the cell cycle and cell proliferation, and the consequences of their misregulation for tumorigenesis, we have only a rudimentary understanding of the spatio-temporal dynamics of tumor cell proliferation in complex 3D systems such as spheroids. Expression of specific cell markers within spheroids can be detected by immunofluorescence microscopy of paraffin-embedded or frozen sections or by using flow cytometry after enzymatic dissociation of the cells, but these methods do not allow us to consider the 3D organization of spheroids[8,9]. A few studies have reported 3D imaging of tumor spheres enriched for cancer stem cells, however, these were small spheres (< 150 μm diameter) comprising few cells [10,11]. The strategies employed cannot be applied to larger spheroids (> 300 μm diameter) that reproduce tumor organization, and they are incompatible with real-time imaging. Indeed, investigation of the dynamics of living cellular processes in 3D inside large spheroids remains technically very challenging.

A light-sheet-based microscopy method known as selective plane illumination microscopy (SPIM) is well adapted to imaging large samples in 3D [12-14]. In SPIM, a sheet of light illuminates the sample perpendicular to the axis of detection at the focal plane of the microscope objective, thus providing optical sectioning of the whole sample. Images are recorded with a CCD camera one plane at a time with high temporal resolution, thus limiting phototoxicity and facilitating imaging of live samples [15]. Furthermore, the combination of several views permits merging of the data obtained at various angles to produce an image in 3D [16]. One study has reported the use of SPIM to image chemically fixed, small (~140 μm diameter) spheroids of BxPC-3 cells - a human pancreatic cancer cell line - stained with the fluorescent DNA dye DRAQ5 [14], reviewed in [17].

In this publication we report the first demonstration that SPIM 3D imaging technology is also adapted to live imaging of MCTS that have been engineered to express fluorescent reporter. It is therefore an attracting new approach to explore unsolved issues on the cell division and proliferation dynamics of cancer cells in 3D using the Multicellular Tumor Spheroid model.

## Results

### Imaging spheroids with a single-plane illumination microscope

In order to investigate, in 3D, cellular processes in large living spheroids, we have built a single-plane illumination microscope in which the light sheet is created by means of a cylindrical lens (Additional file 1). To validate the performance of this setup according to the results obtained by Verveer and collaborators [14], we first performed 3D imaging of fixed large spheroids (> 400 μm diameter) of the human pancreatic cancer cell line Capan-2 stained with DRAQ5 (Additional file 2). An additional movie file shows this in more details (Additional file 3). Lateral and axial resolutions were high enough to identify clearly all the nuclei of the spheroid up to a depth of 200 μm and we were also able to distinguish condensed chromosomes in mitotic cells. We could then visualize the nuclei in the whole volume of the spheroid by rotating the sample in the SPIM, acquiring z-stack images of various views and subsequently merging them (Additional file 2C). These results show that this set-up is well adapted to image, in 3D, individual cells inside spheroids large enough to include cellular heterogeneity along a proliferative gradient distribution.

### Imaging of H2B-HcRed expressing spheroids

**Figure 1:**
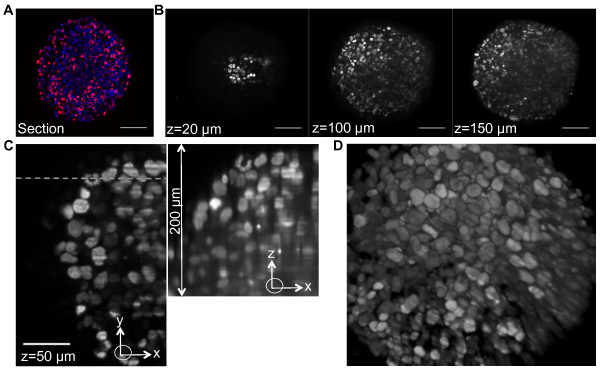
**SPIM imaging of spheroids of HCT116 colon carcinoma cells expressing a histone H2B-HcRed fusion protein**. A: A frozen section from the center of an H2B-HcRed-expressing HCT116 cell spheroid. DNA is stained with DAPI. Scale bar, 100 μm. B: XY planes at the indicated z positions of the 200 μm z-stack shown in Additional file 4 (10× objective, NA = 0.3). C: Region of the XY plane at 50 μm in depth in the z-stack (left). The dashed line indicates the Y position of the XZ plane (right), parallel to the detection axis. Scale bar, 100 μm. D: 3D visualization of the z-stack presented in the Additional file 4.

DRAQ5 DNA staining is unsuited to imaging proliferation of living cells in MCTS because of its poor ability to penetrate deep layers and its toxicity upon long-term incubation. We therefore produced a cell line (HCT116, a human colon carcinoma) stably expressing a fluorescent reporter protein: a fusion of histone H2B with the far-red fluorescent protein HcRed [18]. Expression of fluorescent fusion proteins with H2B has proven useful to study the fates of cells during development [19-21]. The engineered spheroids retained all the biological characteristics of the parental cell line (data not shown). We selected a clone in which only a subpopulation of cells expressed the H2B-HcRed protein in their nuclei (Figure 1A) thus rendering SPIM imaging and segmentation of large H2B-HcRed-expressing spheroids more manageable. The lateral and axial resolution of SPIM allowed us to identify nuclei in these spheroids up to 200 μm in depth (Figure 1). Nuclei in the whole volume of the spheroid could then be visualized by fusion of two stacks at 0° and 180° (Figure 1D, Additional file 4).

### Imaging mitotic cells within spheroids

**Figure 2:**
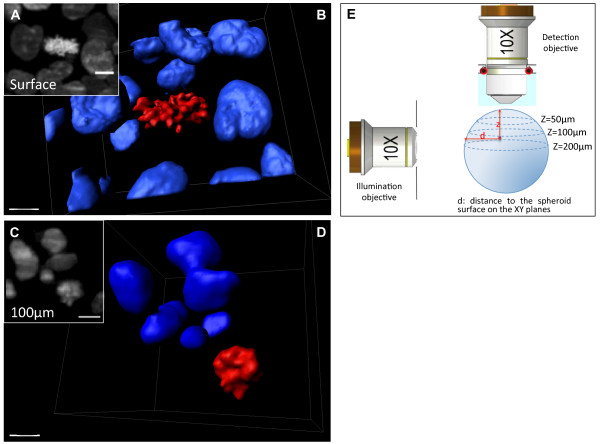
**Imaging and 3D reconstruction of interphase and mitotic nuclei within a fixed H2B-HcRed-expressing spheroid**. A: Magnification of one frame of the z-stack presented in Additional file 4 showing a mitotic cell located on the surface of the spheroid detected at z = 30 μm. Scale bar, 15 μm. B: 3D reconstruction of the region of the stack corresponding to the frame shown in a: blue isosurfaces correspond to interphase nuclei and red isosurfaces to mitotic condensed chromosomes. Scale bar: 7 μm. C: Magnification of a region of a z-stack at z = 150 μm showing a mitotic cell located at 100 μm from the surface of the spheroid. Scale bar, 10 μm. D: 3D reconstruction of the region of the stack corresponding to C. Scale bar: 7 μm. E: Sketch showing how the position of the mitotic cells are defined with regard to the spheroid surface and to the z position in the detection axis.

Mitosis is the phase of the cell cycle when a single cell divides into two daughter cells. During mitosis, the cell's microtubule network is reorganized to form a mitotic spindle that first aligns the duplicated and condensed chromosomes in the centre of the cell and then segregates one set of chromosomes to each pole around which the daughter cell forms. Mitotic cells with condensed chromosomes were easily and clearly identified by SPIM within H2B-HcRed-expressing HCT116 cell spheroids (Figure 2, Additional file 4) by their high fluorescence intensity and the familiar condensed chromosome shape when compared to the round nuclei of the interphase cells (Figure 2A, Additional files 5 and 6). This can be seen in a 3D reconstruction made by using extraction of iso-surfaces of the fluorescence intensity to see individual fluorescent objects in the volume of a region of a z-stack encompassing a mitotic cell (Figure 2B, Additional file 7). These data demonstrate the utility of SPIM imaging for the study of cell division in multicellular 3D tumor models. Numerous gene products involved in progression of mitosis are implicated in tumorigenesis [22]. SPIM provides a means to study the effects of mutations in these genes on, for example, chromosome alignment, the position and orientation of the future cleavage plane and the roles of extrinsic cues such as cell-cell interactions and adhesion to the extracellular matrix taking into account a multicellular context for cancer cells [4,23,24].

### Time lapse imaging of cell division within spheroids

**Figure 3:**
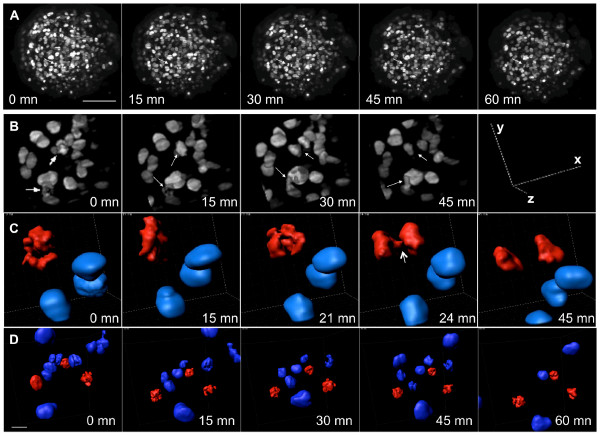
**Live spheroid imaging by SPIM**. A: Maximum projection of z-stacks of an H2B-HcRed-expressing spheroid recorded at the indicated times. The images are taken from Additional file 9. Stacks of 100 slices were recorded by SPIM (10× objective, NA = 0.3) every 3 minutes with a slice spacing of 1 μm. The white arrow shows a dividing cell. Scale bar, 100 μm. B: 3D visualization of an enlarged region of the spheroid at the indicated times. The progress of mitosis can be seen in two cells (arrows). C: 3D reconstruction of the interphase nuclei (blue) and condensed chromosomes (red) of one mitotic cell within the 4D stack. The arrow indicates a sister chromatid disjunction. Scale bar, 10 μm. D: 3D reconstruction of a region of a spheroid treated with paclitaxel showing three mitotic cells (red) blocked in mitosis over 60 minutes. Scale bar, 20 μm.

The low photobleaching and phototoxicity associated with SPIM technology is compatible with imaging living samples of various organisms, as illustrated by the recent work of several groups [15,19,25]. Live imaging experiments using SPIM have been performed previously on Drosophila, zebrafish and sea urchin embryos, which do not require strictly controlled environmental conditions. For many model systems, however, tight control of the physiological environment is crucial for live-cell imaging experiments. To examine whether SPIM could be applied to live 3D imaging studies of cell cycle processes in human MCTS, we adapted our SPIM setup to maintain the temperature at 37°C and the atmospheric CO2 at 5% - the conditions required for spheroid growth - for the duration of the time-lapse experiments. We also developed sample holders made from a biocompatible material with a refractive index close to that of water, in which spheroids can grow without mechanical stress so avoiding the regional effects on cell proliferation that are seen with the agarose-embedding procedure commonly used to present samples for SPIM (Additional file 8)[26,27]. With these improvements, we were able to image in 3D and for several hours H2B-HcRed in the nuclei of cells in spheroids (Figure 3, Additional file 9) and to observe chromosome segregation and cleavage plan orientation (Figure 3, Additional files 10 and 11). Three-dimensional images showing live cell division dynamics and cell behavior in such large multicellular spheroids have not been published previously. These results provide a proof of principle that SPIM can be used to study human cancer cells biology in 3D. Nevertheless, improvements have to be considered concerning the environmental conditions control, sample holding and stack registration over time to optimize the system.

### Application of real time SPIM-imaging to anti-cancer drug activity monitoring

To prove fully the potential of 3D time-lapse SPIM, we monitored the effect of an antimitotic drug, paclitaxel, on mitotic dynamics in spheroids. This microtubule-stabilizing agent is widely used in the clinic [28,29]. Paclitaxel induced a block in mitosis accompanied by continuous dynamic movements of the condensed chromosomes that did not align at the centre of the cell (Figure 3D, additional files 12 and 13). These data demonstrate that 3D dynamic imaging of spheroids by SPIM can be applied to evaluate the activity of putative anti-tumor drugs.

## Conclusions

This study demonstrates the feasibility of using SPIM to observe live cell division dynamics in large spheroids that reproduce the cellular organization in tumor micro-regions including cellular heterogeneity, and cell-cell and cell-microenvironment interactions. SPIM studies of the mechanisms controlling cell proliferation within large spheroids should bring invaluable information that cannot be obtained from 2D, monolayer cell culture models. Furthermore, the strategy reported in this study, together with forthcoming development in image analysis, will be of outstanding interest to the study of systemic cancer biology.

## Methods

### The SPIM setup

Additional file 1 shows the selective-plane illumination microscope used in this work. The setup consisted of two horizontal optical axes perpendicular to each other: a light-sheet illumination axis for selective plane excitation and a detection axis. This homemade SPIM setup was equipped with a compact laser launch (Errol, France) comprising the outputs of three DPSS lasers (491 nm, 532 nm and 595 nm) combined with dichroic mirrors into a single multi-wavelength beam. A four-channel acoustico-optical tuneable filter (AOTF) with a separate blanking channel provided precise control over each laser's illumination intensity. The output of the AOTF was coupled into a fibre. The light sheet was obtained by using a cylindrical lens conjugated to an illumination objective (10× NA 0.25). The sample was positioned in the light sheet inside a chamber filled with aqueous medium. The light was collected by means of an immersion objective (10× NA 0.3). The detection objective was fitted into a physiological chamber connected to a filter wheel and a cooled CCD camera (Roper Scientific). The physiological chamber was manufactured by stereolithography of a photosensitive epoxy resin (Cresilas). Movements of the sample holder (x, y, z and rotation) were performed by a fully motorized stage (Physics Instruments), thus allowing multiview imaging. An incubator was fitted above the instrument to control the temperature and CO2 concentration around the sample (PECON controllers). The whole system was fully controlled by AMISPIM software that we developed specifically for this task by using the Labview development environment. This software allows manual or automated control of all the parameters and devices connected to the instrument (three lasers, motorized stage, camera, shutter, filter wheel, temperature monitoring, CO2 control, data acquisition and management).

### Spheroid production and staining

Capan-2 pancreatic cancer cells were cultured in DMEM/F12 (Invitrogen, France) containing 10% FCS with 2 mmol/l glutamine and 1% penicillin/streptomycin in a humidified atmosphere of 5% CO2 at 37°C. Spheroids were prepared as previously described [30]. Briefly, 100 μl of a suspension of 10,000 cells/ml in DMEM/F12 supplemented with 2 nM EGF (Invitrogen) and 2% B27 (Invitrogen) was placed in each well of poly-HEMA-coated 96-well plates (10 mg/ml, Sigma). The plates were centrifuged at 200 g for 6 min and then incubated at 37°C. The diameter of the spheroids was measured with a calibrated eyepiece reticule. When the spheroids reached 400 μm in diameter, they were rinsed with PBS and fixed in 4% neutral-buffered formalin (Sigma) for 2-20 h then incubated in DRAQ5 (Ozyme, 1:1000) diluted in PBS, in the presence of RNAse A (Sigma)(1 mg/ml).

We constructed a pcDNA3 plasmid encoding an H2B-tandem HcRed fusion protein and a hygromycin resistance gene. This plasmid was transfected in HCT116 cells (Jet PEI reagent, Polyplus Transfection). One day after transfection, hygromycin (0.2 mg/ml) was added to select cells that had stably incorporated the plasmid. Spheroids from this cell line were obtained as described above.

### Fixed sample mounting for SPIM

The spheroid was placed in 1% low-melting-point agarose cooled to 40°C. This mixture was drawn into a 50 μl glass capillary (Sigma) and cooled for few minutes at 4°C. The capillary was then fixed onto the theta-stage and placed in the PBS-filled physiological chamber (Additional file 1). The column of agarose was then extruded from the capillary with an adapted plunger and the agarose-embedded sample was placed in front of the objective lens.

### Sample holder preparation for time-lapse data acquisition

Sample holders were made from Phytagel™ (also known as Appliedgel or Gelrite, Sigma), an anionic linear tetrasaccharide that polymerizes into a gel in the presence of salt ions. A 10 g/l solution of Phytagel was prepared in PBS and autoclaved. Prior to use, the Phytagel solution was melted in a microwave then used to fill a tip-less Combitip syringe (1250 μl, Gilson) as described in Additional file 8. The molded chamber obtained was then filled with Optimem (Invitrogen) culture medium containing 10% FCS and 1% penicillin/streptomycin. The spheroid was placed in the molded chamber then fixed to a Combitip from which the tip had been excised (Additional file 8). More information is available at http://www.ip3d.fr/IP3D/SPIM/SPIM.html. This assembly was then placed in the physiological chamber filled with Optimem culture medium containing 10% FCS and penicillin/streptomycin. Prior time-lapse acquisition, the physiological chamber was soaked in bleach solution in order to avoid any contamination. To study the effect of taxol treatment, spheroids were incubated in medium containing 100 nM Paclitaxel (Sigma) for a few hours before time-lapse data acquisition then transferred to sample holders filled with medium containing 100 nM Paclitaxel. The physiological chamber was also filled with the same medium.

### Data acquisition

A 10× NA 0.3 water immersion lens (Leica) was used to provide suitable working distances for imaging entire spheroids and 3D resolution sufficient to see individual nuclei. Exposure times were 200-500 ms. DRAQ5 or HcRed fluorescence was excited with a 595 nm laser and detected by using a 593 long-pass filter. The voxel size was 0.645 × 0.645 × 1 μm. Live imaging was performed at 37°C and 5% CO2.

### Image processing

Images were processed with the open-source image-processing package Fiji software. Multiviews registration and fusion were performed using the SPIM registration plugin (Additional file 14) [16]. 3D visualization of the fused stacks was obtained by using the 3D stitching and 3D viewer plug-ins. Drift that occurs between the different time points during time-lapse acquisition was compensated by using the TJ shift transform plug-in.

To perform 3D reconstruction, the stripes that impair SPIM images were first removed using a dedicated algorithm. The stripes are modelled as the convolution product of a white noise by an elementary stripe-like pattern. The images are then denoised using a Maximum A Posteriori model, leading to a nonlinear optimization problem. This software is available on demand. The 3D reconstruction of mitotic cells was performed with the IMARIS 7.0.0 software (Bitplane).

## Competing interests

The authors declare that they have no competing interests.

## Authors' contributions

CF, VL and JF carried out imaging experiments. GG contributed the SPIM microscope assembly. AM developed and wrote the AMISPIM software. JF and PW developed dedicated imaging processing.

CL, VL and BD conceived the study, and participated in its design and coordination and helped to draft the manuscript. All authors read and approved the final manuscript.

## Supplementary Material

Additional file 1 **The Selective Plane Illumination Microscopy (SPIM) setup**. A: Schematics of the SPIM system. Lateral (B) and top (C) views of the SPIM setup. The green path corresponds to the illumination axis and the perpendicular red path corresponds to the detection axis. The sample is suspended through the theta-stage (θS) in the physiological chamber (PC) and is positioned in the focal plane of the detection objective. It can be moved in the x, y and z axis and rotated (θ axis, blue arrow in c). T: telescope; M: mirror; CL: cylindrical lens; TL: tube lens; IO: illumination objective; PC: physiological chamber; XS: X-stage; YS: Y-stage; ZS: Z-stage, θS: θstage; F and FW: filter wheel. D: The physiological chamber was designed using the Open source Blender software (top) and then manufactured by stereolithography of a photosensitive epoxy resin (bottom). The two tinny holes on the top of the chamber (red circles) allow to inject CO2 directly in the physiological chamber at the surface of the culture medium. Click here for file

Additional file 2 **SPIM images of a spheroid of Capan-2 human pancreatic cancer cells labelled with DRAQ5™**. A: Raw images corresponding to the XY optical sections at the indicated depths inside the spheroid. Scale bar 50 μm. The white arrows show dividing cells located at several cell layers of depth inside the spheroid. B: The XY plane at 130 μm depth is shown with the XZ and ZY planes, parallel to the detection axis, at the Y and X positions indicated by the dashed lines (Scale bar 50 μm). The inserts correspond to the enlargement of the region in the white square on the XY section that displays a mitotic cell. Scale bar 5 μm. The progressive loss of signal observed along the x-axis results from light scattering and absorption by the spheroid that attenuates the light sheet illumination. Horizontal stripes parallel to the light sheet (x-axis) are sample-dependent artifacts specific to SPIM technology. C: 3D visualisation of a multiview reconstruction of four stacks recorded at various angles (0-315°) at incremental steps of 90°. The corresponding stack is shown in Additional file 14. Click here for file

Additional file 3 **Three-dimensional SPIM imaging of a Capan-2 cell spheroid labelled with DRAQ5™**. This movie shows a z-stack of 250 slices at a slice spacing of 1 μm. Arrows show mitotic cells. Laser 595 nm; illumination objective 10× NA = 0.25; detection objective 10× NA = 0.3. Scale bar 50 μm. Click here for file

Additional file 4 **Three-dimensional SPIM imaging of an H2B-HcRed-expressing spheroid**. Movie showing two merged z-stacks at 0° and 180°. For each z-stack, 200 slices were recorded with a slice spacing of 1 μm. Arrows show mitotic cells. Click here for file

Additional file 5 **Three-dimensional SPIM imaging of mitoic cell inside an H2B-HcRed-expressing spheroid**. This movie shows 28 slices through a mitotic cell. The images correspond to a region from the z-stack shown in Additional file 4. Click here for file

Additional file 6 **Three-dimensional visualization of two regions of the stack shown in Additional file**5. Arrows show mitotic cells. This visualization was obtained with the 3D viewer plug-in of Fiji software. Click here for file

Additional file 7 **3D reconstruction of a mitotic cell inside an H2B-HcRed-expressing spheroid**. Visualization of the 3D reconstruction of the stack shown in the right part of the Additional file 6 (blue isosurfaces, interphase nuclei; red isosurfaces, mitotic condensed chromosomes). Click here for file

Additional file 8 **Sample holder preparation for time-lapse acquisitions**. A: Sample holders were prepared using a 1.25 ml Combitip from which the tip has been removed. A Phytagel solution (10 g/l in PBS) is aspirated in the Combitip and formed after polymerisation the sample holder. B: The sample holder (shaded grey) was uncast by applying gentle pressure then suspended on a plunger for transfer into the physiological chamber of the microscope. The plunger was made from a Combitip with the tip cut off to leave an empty space (light grey). C: Enlargement of the Phytagel sample holder showing the cavity generated by the shape of the tip of the Combitip plunger. Culture medium was placed in this cavity, in which a spheroid can grow. For more details on sample holder preparation an illustrated protocol is available for downloading here: http://www.ip3d.fr/IP3D/SPIM/SPIM.html Click here for file

Additional file 9 **SPIM imaging of a live spheroid**. SPIM imaging of a live spheroid expressing H2B-HcRed. Z-stacks of 100 slices at slice spacing of 1 μm were recorded every three minutes (10× objective, NA = 0.3). The maximum projection of the z-stacks is shown for each time point. Click here for file

Additional file 10 **3D visualization of the division of two cells**. Three-dimensional visualization of an enlarged region of Additional file 9 showing the progress of two cells through mitosis. This visualization was obtained with the 3D viewer plug-in of Fiji software. Arrows show the two dividing cells. Click here for file

Additional file 11 **3D reconstruction of the division of two cells**. Visualisation of the isosurfaces corresponding to the 3D reconstruction of the mitotic chromosomes (red) and interphase nuclei (blue) in a region of the stack shown in Additional file 9. Click here for file

Additional file 12 **SPIM imaging of a live H2B-HcRed-expressing spheroid treated with Paclitaxel**. Z-stacks of 200 slices at slice spacing of 1 μm were recorded every three minutes. For each time point, the maximum projection of an enlarged region of each stack is shown (10× objective, NA = 0.3). Arrows indicate mitotic cells. Click here for file

Additional file 13 **3D reconstruction of arrested mitotic cells following taxol treatment**. Visualisation of the isosurfaces corresponding to the 3D reconstruction of a region of the stack shown in Additional file 12. Click here for file

Additional file 14 **Stack of the registered and fused multiviews through a spheroid stained with DRAQ5**. Single planes of the stack of the reconstructed spheroid showed in the Additional file 2C. Click here for file
