# Supplementary figures and images for: Live cell division dynamics monitoring in 3D large spheroid tumor models using light sheet microscopy
Source: Cell Div. 2011 Dec 12;6:22. doi: 10.1186/1747-1028-6-22 (PMC3274476; doi:10.1186/1747-1028-6-22)

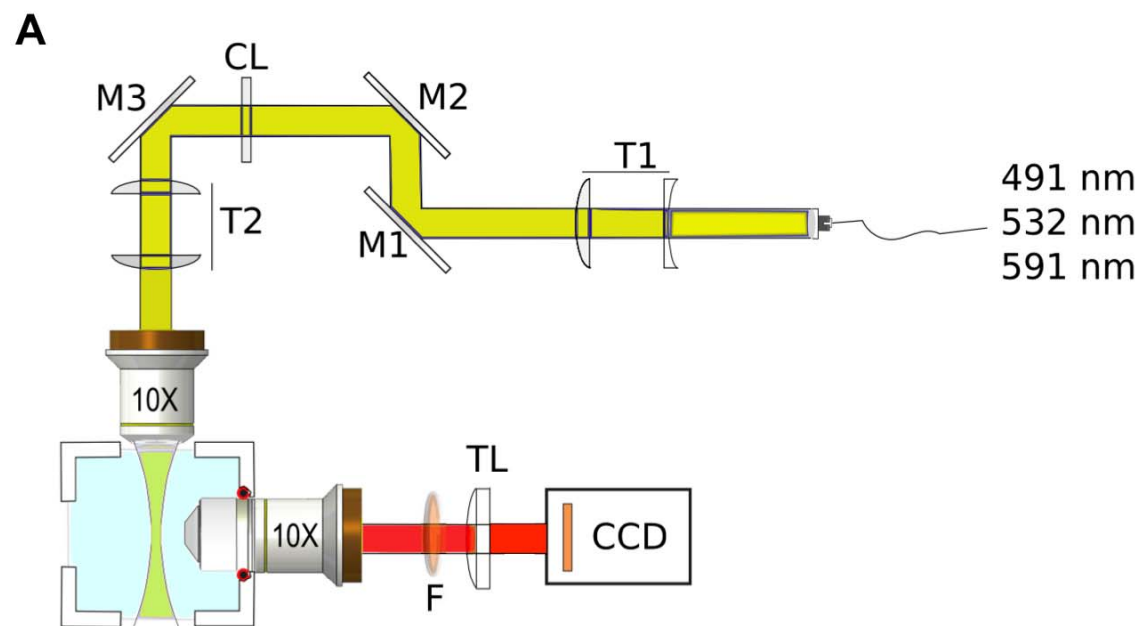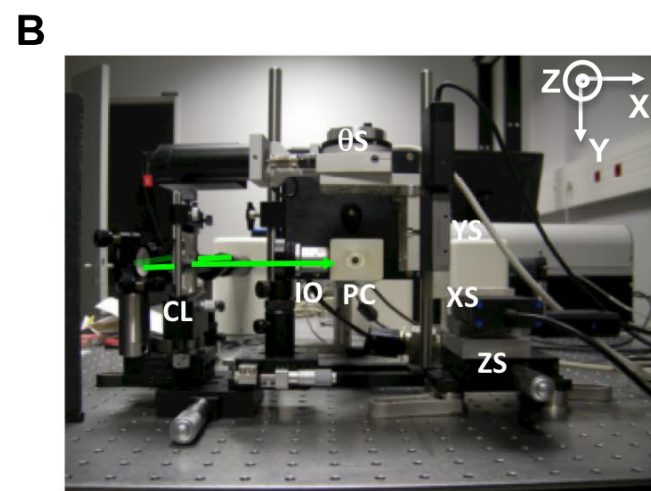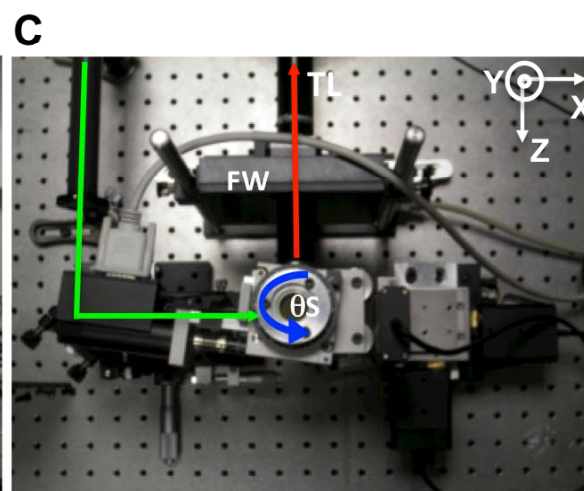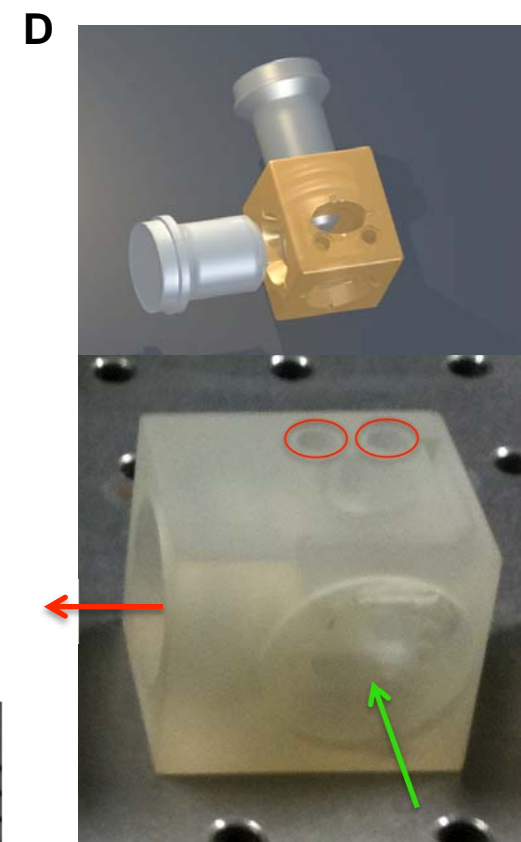

Supplement: Additional file 1 — The Selective Plane Illumination Microscopy (SPIM) setup. A: Schematics of the SPIM system. Lateral (B) and top (C) views of the SPIM setup. The green path corresponds to the illumination axis and the perpendicular red path corresponds to the detection axis. The sample is suspended through the theta-stage (θS) in the physiological chamber (PC) and is positioned in the focal plane of the detection objective. It can be moved in the x, y and z axis and rotated (θ axis, blue arrow in c). T: telescope; M: mirror; CL: cylindrical lens; TL: tube lens; IO: illumination objective; PC: physiological chamber; XS: X-stage; YS: Y-stage; ZS: Z-stage, θS: θstage; F and FW: filter wheel. D: The physiological chamber was designed using the Open source Blender software (top) and then manufactured by stereolithography of a photosensitive epoxy resin (bottom). The two tinny holes on the top of the chamber (red circles) allow to inject CO2 directly in the physiological chamber at the surface of the culture medium. [file 1747-1028-6-22-S1.PDF]

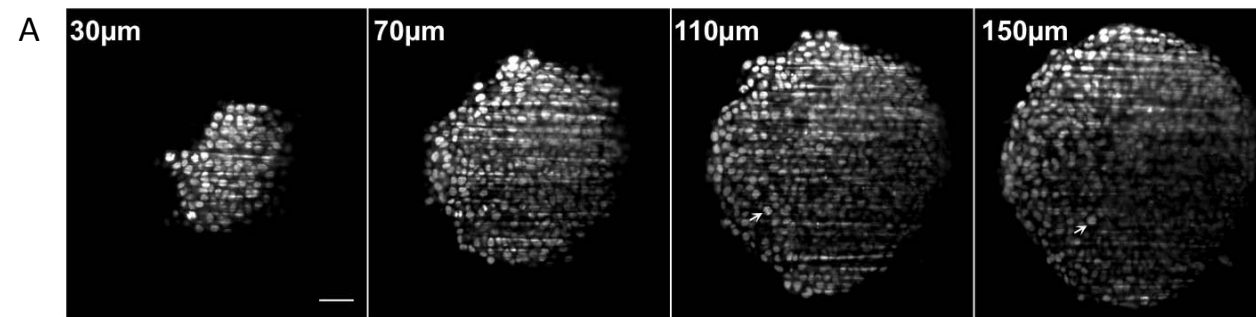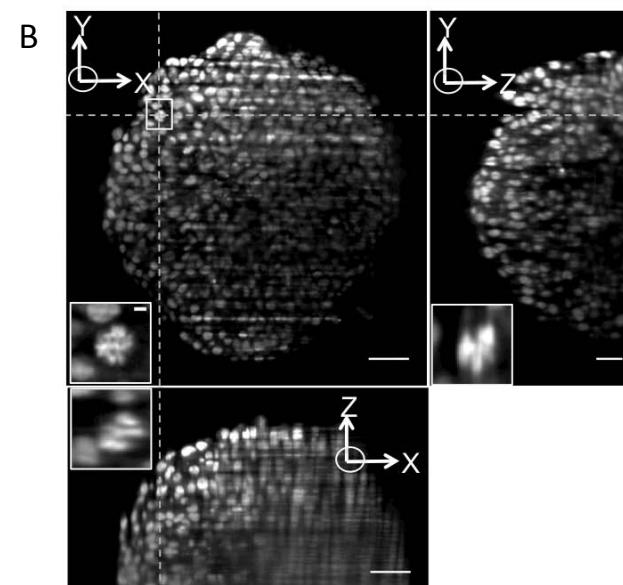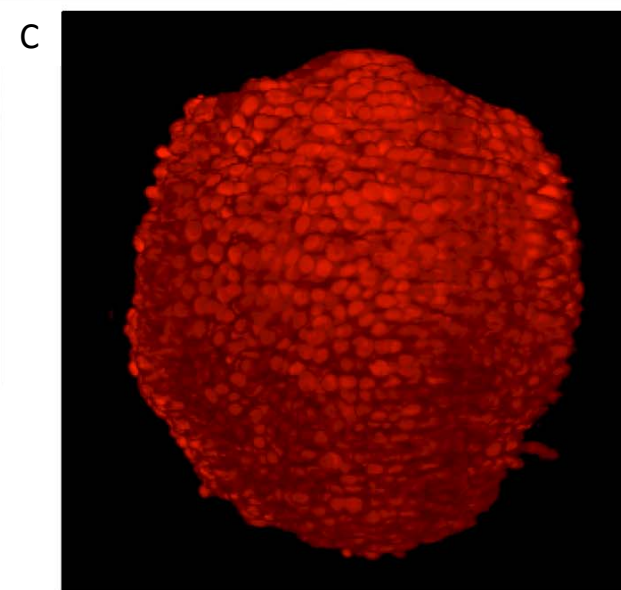

Supplement: Additional file 2 — SPIM images of a spheroid of Capan-2 human pancreatic cancer cells labelled with DRAQ5™. A: Raw images corresponding to the XY optical sections at the indicated depths inside the spheroid. Scale bar 50 μm. The white arrows show dividing cells located at several cell layers of depth inside the spheroid. B: The XY plane at 130 μm depth is shown with the XZ and ZY planes, parallel to the detection axis, at the Y and X positions indicated by the dashed lines (Scale bar 50 μm). The inserts correspond to the enlargement of the region in the white square on the XY section that displays a mitotic cell. Scale bar 5 μm. The progressive loss of signal observed along the x-axis results from light scattering and absorption by the spheroid that attenuates the light sheet illumination. Horizontal stripes parallel to the light sheet (x-axis) are sample-dependent artifacts specific to SPIM technology. C: 3D visualisation of a multiview reconstruction of four stacks recorded at various angles (0-315°) at incremental steps of 90°. The corresponding stack is shown in Additional file 14. [file 1747-1028-6-22-S2.PDF]

A

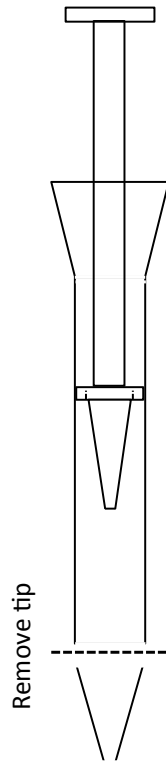

B

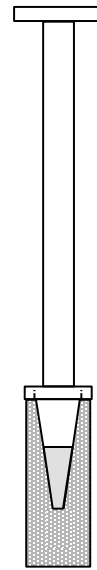

C

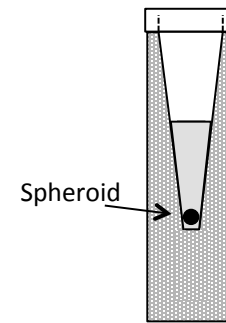

Supplement: Additional file 8 — Sample holder preparation for time-lapse acquisitions. A: Sample holders were prepared using a 1.25 ml Combitip from which the tip has been removed. A Phytagel solution (10 g/l in PBS) is aspirated in the Combitip and formed after polymerisation the sample holder. B: The sample holder (shaded grey) was uncast by applying gentle pressure then suspended on a plunger for transfer into the physiological chamber of the microscope. The plunger was made from a Combitip with the tip cut off to leave an empty space (light grey). C: Enlargement of the Phytagel sample holder showing the cavity generated by the shape of the tip of the Combitip plunger. Culture medium was placed in this cavity, in which a spheroid can grow. For more details on sample holder preparation an illustrated protocol is available for downloading here: http://www.ip3d.fr/IP3D/SPIM/SPIM.html [file 1747-1028-6-22-S8.PDF]
